# miRIAD—integrating **mi**cro**R**NA **i**nter- **a**nd intragenic **d**ata

**DOI:** 10.1093/database/bau099

**Published:** 2014-10-04

**Authors:** Ludwig Christian Hinske, Gustavo S. França, Hugo A. M. Torres, Daniel T. Ohara, Camila M. Lopes-Ramos, Jens Heyn, Luiz F. L. Reis, Lucila Ohno-Machado, Simone Kreth, Pedro A. F. Galante

**Affiliations:** ^1^Clinic of Anaesthesiology, Clinic of the University of Munich, Munich, Germany; ^2^Centro de Oncologia Molecular, Hospital Sírio-Libanês, São Paulo, SP 01308-060, Brazil; ^3^Departamento de Bioquímica, Instituto de Química, Universidade de São Paulo, São Paulo, Brazil; ^4^Division of Medial Informatics, University of California San Diego, La Jolla, CA 93093-0505, USA

## Abstract

MicroRNAs (miRNAs) are a class of small (∼22 nucleotides) non-coding RNAs that post-transcriptionally regulate gene expression by interacting with target mRNAs. A majority of miRNAs is located within intronic or exonic regions of protein-coding genes (host genes), and increasing evidence suggests a functional relationship between these miRNAs and their host genes. Here, we introduce miRIAD, a web-service to facilitate the analysis of genomic and structural features of intragenic miRNAs and their host genes for five species (human, rhesus monkey, mouse, chicken and opossum). miRIAD contains the genomic classification of all miRNAs (inter- and intragenic), as well as classification of all protein-coding genes into host or non-host genes (depending on whether they contain an intragenic miRNA or not). We collected and processed public data from several sources to provide a clear visualization of relevant knowledge related to intragenic miRNAs, such as host gene function, genomic context, names of and references to intragenic miRNAs, miRNA binding sites, clusters of intragenic miRNAs, miRNA and host gene expression across different tissues and expression correlation for intragenic miRNAs and their host genes. Protein–protein interaction data are also presented for functional network analysis of host genes. In summary, miRIAD was designed to help the research community to explore, in a user-friendly environment, intragenic miRNAs, their host genes and functional annotations with minimal effort, facilitating hypothesis generation and *in-silico* validations.

**Database URL:** http://www.miriad-database.org

## Introduction

Amongst regulatory mechanisms of gene expression in eukaryotes, microRNAs (miRNAs) have established a central role in the past two decades (1). These 22-nt short single-stranded RNA molecules guide the RNA-induced silencing complex to modulate the expression of target mRNAs (2). MicroRNA binding sites are most likely recognized by nucleotide sequences in the 3'-untranslated regions (3′-UTR) of target mRNAs. Binding of the miRNA–protein complexes to their targets results in either degradation or translational inhibition of the mRNA transcripts (2).

For humans, ∼1900 miRNA genes have been identified (3), and more than half are located within genomic regions containing protein-coding genes (4–6). Hence, miRNA genes can be classified as either inter- or intragenic, and the latter sub-classified as intronic or exonic (4, 5). A substantial number of these intragenic miRNAs are co-transcribed, and consequently co-regulated with their host genes (4, 5, 7). Recent evidence suggests a functional linkage between intragenic miRNAs and their hosts on multiple levels, including direct and indirect interaction (8–10).

Despite the importance of these intragenic miRNAs, their exploration can be daunting, as much of the necessary information is not readily available and requires manual integration from multiple data sources (6, 11, 12). Although other databases exist that provide information related to intra- and intergenic miRNAs (12–15), some tools don’t appear to be frequently updated (14), contain only an elementary set of information related to intragenic miRNAs and their host genes (13, 15) and/or their usage is complex and requires in-depth bioinformatics skills (12).

In the current manuscript, we present miRIAD, a web-service designed to examine intragenic miRNAs, their host genes and their functional annotations with a streamlined graphical data representation and an efficient information query system. miRIAD provides information regarding genomic context, gene function, gene interaction, miRNA targets and gene expression for five species, including human and mouse. miRIAD is publicly available at http://www.miriad-database.org.

## Materials and Methods

### Database architecture and raw data

Because miRIAD integrates a large set of data, processed information is stored in a MySQL relational database. Supplementary Figure S1 provides an overview of the miRIAD database schema, its tables and their relations. To date, miRIAD consists of 60 tables in total, comprising 12 tables for each of the five species (human, rhesus monkey, mouse, opossum and chicken), containing ∼10 million records of integrated information.

To construct miRIAD, we used several sets of publicly available data. The reference genomes (human genome sequence—GRCh37/hg19; rhesus genome—rheMac3; mouse genome—mm10/GRCm38; opossum genome—MonDom5; chicken genome—galGal4) were downloaded from UCSC Genome Browser (http://genome.ucsc.edu). The transcriptome sets were downloaded from the RefSeq project (http://www.ncbi.nih.gov/refseq) for all species. MicroRNA genomic coordinates, seed sequences and family information were retrieved from miRBase (http://www.mirbase.org/, release #20). Protein–protein interaction data were acquired from the Human Protein Reference Database (HPRD, downloaded from NCBI http://www.ncbi.nih.gov) and from EMBL’s STRING database (http://string-db.org/). Gene expression data were obtained from Brawand *et al.* (16) (coding genes) and Meunier *et al.* (17) (miRNAs).

### Host gene and miRNA information

All known genes were classified either as host or non-host based on the presence of overlapping miRNAs for each species. This classification and additional information regarding known genes were stored in three tables (GeneInformation, GeneRegions and GeneSynonyms), as shown in Supplementary Figure S1.

All miRNA genes were classified either as intra- or intergenic, based on their genomic localization. The ‘MirnaInformation’ table contains the official name, genomic coordinates of the stem loop sequence and, if applicable, the host gene to which the miRNA is related. In case of multiple genes, the host gene assigned was the one on the same strand as the miRNA. If intronic, the intron number and the region length between the miRNA coordinates and the next exon upstream were calculated and stored.

### miRNA target prediction

miRIAD contains all conserved target sites within 3’UTRs from TargetScan (http://www.targetscan.org/, release #6.2) for human and mouse. In brief, TargetScan defines miRNA targets by searching, within 3’ UTR regions, for 8mer (exact match) and 7mer sites that match the seed region (position 2–7) of mature miRNAs. Information regarding interspecies conservation and match/mismatch profile are also used to define the final set of conserved targets (for further information, see http://www.targetscan.org/). miRIAD contains a total of 1141 miRNAs binding to 466569 mRNA targets from 14867 known protein coding genes for human. Target prediction information for human and mouse were directly downloaded from the TargetScan homepage (file Conserved_ Site_Context_ Scores.txt, release #62) and calculated for rhesus monkey, opossum and chicken miRNAs using the TargetScan tool kit, applied to all miRNAs and the 3’UTRs from these organisms.

### Gene and miRNA expression

To obtain expression for protein-coding genes, data from Brawand *et al.* (16) were downloaded from GEO (GSE30352) and aligned to the genome of each species using TopHat (version 2.0.8b) with default parameters (18). Normalized gene expression values for six tissues (brain, cerebellum, heart, liver, kidney and testis) from all species were computed by means of FPKM (19) with Cufflinks [version 2.2.1; (20)] using transcript annotations from Ensembl (version 71). To determine miRNA expression available for five tissues (brain, cerebellum, heart, kidney and testis) from all species, data from Meunier *et al.* (17) were downloaded from GEO (GSE40499) and reads were aligned to each genome with Bowtie version 1.0.0 using the following parameters: -m 5 -v 0 -a –best –strata. Only exact matches were considered, and reads aligned to >5 different loci were discarded. The 3’ adaptors were removed using a sequential trimming strategy (21). Reads totally overlapping to mature miRNA coordinates annotated from miRBase (release 20) were counted and normalized for each species with EdgeR package version 2.6.12 (22). Host gene and intragenic miRNA expression correlations were calculated by Spearman’s rank correlation using the normalized values (FPKM and CPM (counts per million) for coding genes and miRNAs, respectively).

## Results

### Database overview

**Figure 1.**
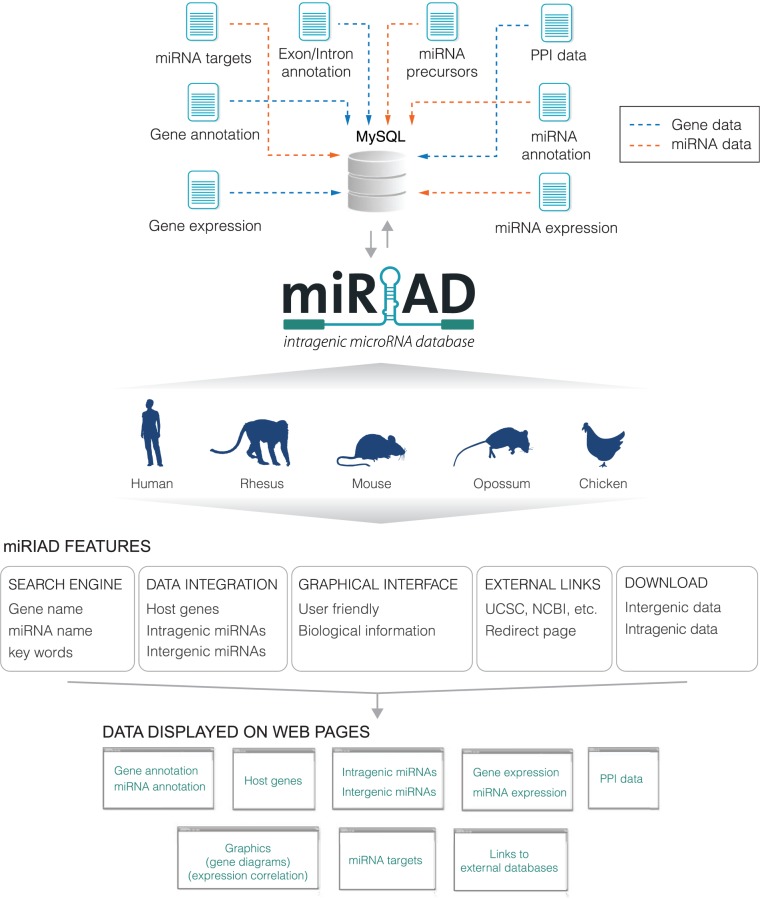
Overview of the miRIAD platform. Schematic representation of the main data presented in the web tool and how they are integrated and displayed. Blue arrows denote data related to protein-coding genes and orange arrows indicate data related to miRNAs. PPI: protein–protein interaction.

Figure 1 summarizes the main features, data sets and how information is presented in the miRIAD web tool. Most of miRIAD data related to intragenic miRNAs and their host genes is summarized in Table 1. To provide a useful platform, miRIAD integrates all known protein-coding genes (∼22k genes on average, for all five species), all known miRNAs (∼900 on average, for all five species), miRNAs targets, validated and predicted protein–protein interactions and expression data for miRNAs and coding genes across five and six tissues, respectively. miRIAD classifies all miRNAs as intragenic or intergenic. It contains a total of 1072 (57%) for human; 167 (29%) for rhesus; 745 (63%) for mouse; 179 (40%) for opossum; 299 (52%) for chicken, additionally specifying whether or not they are transcribed in the same orientation as that of their host genes (84, 54, 87, 92 and 76% of intragenic miRNAs for human, rhesus, mouse, opossum and chicken, respectively). It is worth mentioning that some of the discrepancies between these percentages are likely due to the incompleteness of miRNA and gene annotation for individual species. As we can observe for human and mouse, which have the most complete annotated sets of coding genes and miRNAs, the values are quite similar. Additional complex information is also provided, such as the visualization of intragenic miRNAs within their host genes and positioning along the isoforms, expression correlation between intragenic miRNAs and their host genes, intragenic miRNAs binding to their own host genes and intragenic miRNAs binding to genes that are directly interacting with their host genes. These data are necessary in the identification and evaluation of putative negative or positive feedback mechanisms between miRNAs and host genes, (5, 23–25) and can offer a starting point for future analyses to reveal novel regulatory pathways.

**Table 1.** Summary of main miRIAD data

Data class	Human	Rhesus	Mouse	Opossum	Chicken
Known protein-coding genes	20 530	22 553	29 664	20 550	16 953
Known miRNA precursors	1871	582	1181	443	573
Intragenic miRNAs	1072	167	745	179	299
Intergenic miRNAs	799	415	435	264	272
Host genes	930	141	613	143	273
Sense miRNAs in respect to host orientation	902	90	645	145	90
Antisense miRNAs in respect to host orientation	170	77	95	12	28
Expressed coding genes	18 442	8112	19 029	12 079	11 278
Expressed miRNAs	1111	475	784	405	465

### miRIAD query system

The miRIAD query system was developed and optimized to be fast, intuitive and functional. It lets the user search for several terms, such as miRNA symbol, gene name (Official Symbol, Ensembl ID, Entrez ID, HGNC ID or Gene Synonyms) and gene annotation keywords (e.g. ‘oncogene’, ‘kinase’, etc.). Searching for miRNAs follows the same principles as those used for coding genes, allowing for non-exact inputs (according to miRNA official nomenclature). It is also possible to query for multiple genes or miRNAs at once. The query system works in the same way for all five species.

The output for each searched term is a list of query matches organized by relevance, containing basic gene information for rapid inspection and selection. Names of host genes and intragenic miRNAs are readily identified by a particular tag (see web page for details). Moreover, non-host genes and intergenic miRNAs are also shown, because they may have indirect associations to intragenic miRNAs or host genes and are therefore also important. By clicking on a gene name, the user can access more detailed information about any known coding or miRNA gene.

### Exploring host genes

**Figure 2.**
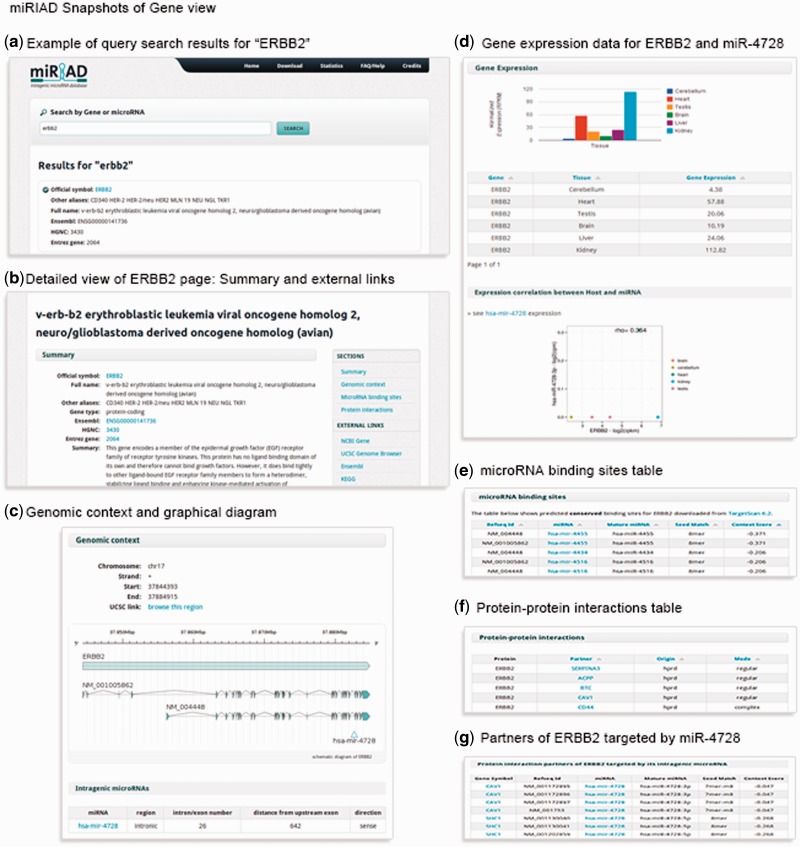
A summary of the main information presented in miRIAD for the coding gene ERBB2 and its intragenic mir-4728.

In the recent past, it has become clear that functional aspects of intragenic miRNAs have to be viewed in the context of their host genes (5, 7, 23, 24, 26). Therefore, information about all known protein-coding genes has been integrated into miRIAD to allow contextual search. For each protein-coding gene, miRIAD provides a ‘Summary’ section showing annotation data, such as official gene symbol, full gene name and gene name aliases, gene type and a gene function summary when publicly available. Moreover, information regarding the genomic context, including the genomic position, transcription ‘start’ and ‘end’ and transcription orientation, is provided, as well as a graphical representation of the exon–intron structure of transcripts (Figure 2). If applicable, miRIAD presents miRNA name, genomic region (intronic/exonic), the intron/exon number where they are inserted, the distance to the closest upstream exon and transcriptional orientation, sense (miRNA and host in the same transcriptional orientation), or antisense (in opposite orientation). To facilitate the generation and evaluation of research hypotheses, expression data (based on RNA-Seq) of mRNAs across six tissues (brain, cerebellum, heart, kidney, liver and testis) as well as expression correlation between host genes and their intragenic miRNAs were included. All miRNAs potentially binding to a target gene are displayed under ‘miRNA binding sites’. Finally, the last section shows all known protein–protein interaction data for each gene. Cases in which interaction partners of a given host gene are targeted by its intragenic miRNA are explicitly shown. This kind of information is noteworthy because it can reveal unusual regulatory loops and may support findings or suggest future investigations. All these information are exemplified for the oncogene ERBB2 containing mir-4728 (Figure 2).

The gene section also provides links to external databases, such as NCBI Gene (http://www.ncbi.nlm.nih.gov/gene), UCSC Genome Browser (http://genome.ucsc.edu/), Ensembl (http://www.ensembl.org/), KEGG (http://www.genome.jp/kegg/) and Targetscan (http://www.targetscan.org/). Most of these links are context-sensitive, easily redirecting the user to the gene of interest on the web page containing complementary data.

### Intragenic miRNAs

**Figure 3.**
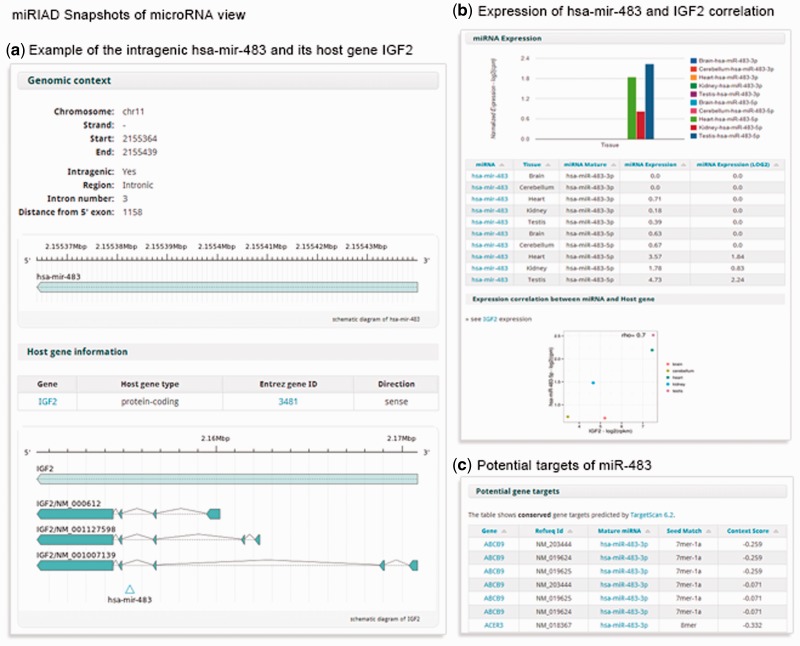
A summary of the main information presented in miRIAD for the intragenic mir-483 and its host gene IGF2.

Intragenic miRNAs are the main focus of our web tool, even though we present information for all known miRNAs and protein-coding genes. For each pre-miRNA, miRIAD provides a ‘Summary’ section with the official miRNA symbol, its full name, miRBase ID, target genes and the genomic context where each miRNA is mapped (Figure 3). For intragenic miRNAs, information about their intragenic position and location along the host genes are depicted by a graphical representation (Figure 3). Cases where an intragenic miRNA potentially targets its own host are highlighted for fast identification. Similar to the presentation of information about protein-coding genes, there are also expression data (based on RNAseq) for six tissues (brain, cerebellum, heart, kidney, liver and testis) and an expression correlation between intragenic miRNAs and their host genes. A set of context-sensitive links to external databases in the top right corner to access complementary information (miRBase, miRDB, Targetscan, mirgen, Magia, miRWalk and miRò) are also presented.

Figure 3 exemplifies the use of this information for mir-483 and its host IGF2. IGF2 produces the insulin-like growth factor 2, an essencial protein for growth and development of the fetus and it is upregulated in several malignancies (27). According to our data, the expression of IGF2 and miR-483-5p are highly correlated (rho = 0.7). Accordingly, a recent report has uncovered a positive feedback between IGF2 and its intragenic mir-483, where the mature miR-483-5p molecule binds to the 5’UTR of IGF2 mRNA, promoting IGF2 transcription by facilitating the association of the helicase DHX9 (24).

### Using miRIAD to explore a set of genes

In the following paragraph, we briefly illustrate how miRIAD can be used to explore a gene or a set of genes. Recently, da Cunha *et al*. (28) defined the set of all human genes coding for cell surface proteins (called surfaceome genes). These genes can be considered as potential targets for diagnostic and therapeutic interventions (28, 29).

The set of 3702 human surfaceome genes was retrieved from (28, 29) and submitted to miRIAD to initially be classified as host or non-host genes. In total, 119 surfaceome genes are host genes for 150 intragenic miRNAs. Interestingly, most of these miRNAs (87.3%) are transcribed on the same orientation of their host genes, suggesting possible co-transcription (5). 140 of these intragenic miRNAs are actually inserted within intronic regions of surfaceome genes.

Next, we examined two genes in more detail. We selected the genes containing the largest number of intronic miRNAs, CLCN5 and HTR2C. In respect to CLCN5, mutations in its sequence have been proven to be associated with diseases of renal tubules, resulting in chronic renal failure (30). This gene has eight intronic miRNAs, and surprisingly, some of their transcripts may be targeted by their intronic miR-502 (see miRIAD).

It is also striking that this host gene has isoforms starting transcription upstream of the miRNAs, which possibly could prevent co-expression between a CLCN5 transcript and those intronic miRNAs in some tissues or pathologies. Analysis of the expression data suggests co-expression or at least co-regulation between CLCN5 and its intronic miRNAs. CLCN5, as well as its intronic miRNAs are highly expressed in kidney. The expression correlations are high (rho > 0.7, Spearman’s rank correlation) for most of the intragenic miRNAs. The functional relationships between CLCN5 and its intronic miRNAs have not been explored yet, though, and deserve further exploration. Suggesting a conserved regulation, a similar pattern is found for Clcn5 gene in mouse, which has five annotated intragenic miRNAs and also a high expression correlation between miRNAs and the host gene.

**Figure 4.**
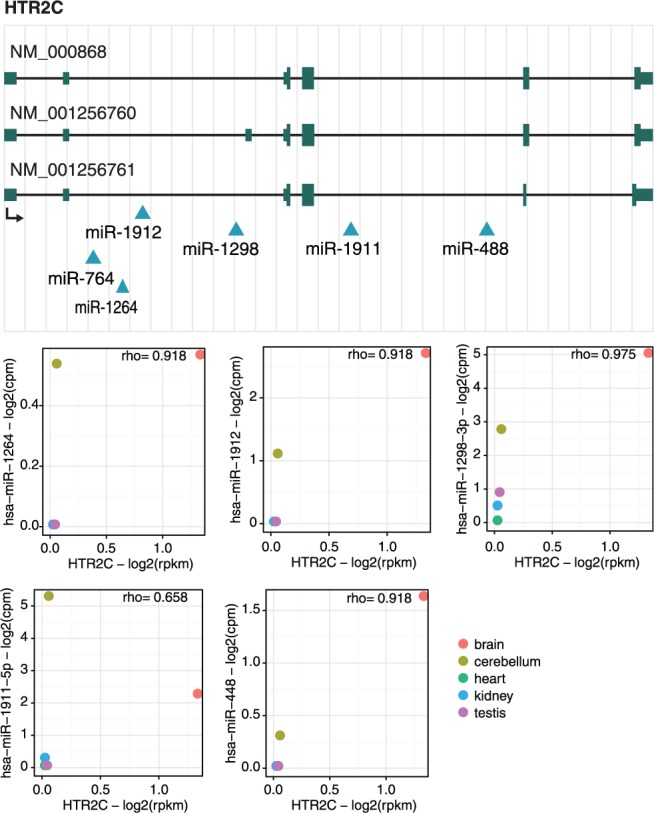
HTR2C gene locus. Genomic mapping of HTR2C transcripts (NM_000868, NM_001256760 and NM_001256761) and their six intragenic miRNAs (miR-1912, miR-764, miR-1264, miR-1298, miR-1911 and miR-488) as well as the expression correlation between HTR2C and these miRNAs. The diagram represents the gene structure according to UCSC genome browser. Because the expression of miR-764 could not be detected, expression correlation for this miRNA and its host gene is not shown.

The second gene, HTR2C, encodes the 2C subtype of serotonin receptor and contains six intronic miRNAs (Figure 4). Similar to CLCN5, host and miRNAs have the same transcriptional orientation (see miRIAD web page for details). As reported by (10), up-regulation of HTR2C is involved in adipocyte differentiation by repressing the KLF5 gene through the expression of miR-448, a miRNA located in the fourth intron of HTR2C. Interestingly, our expression data show a highly positive (rho > 8.5, Spearman’s rank correlation) correlation between miRNAs and host gene, being expressed specifically in cerebellum and brain (Figure 4). The patterns of co-expression are also conserved in opossum and mouse. Moreover, HTR2C is tightly involved in important neuropsychiatric disorders (31); thus, the functional consequences of the concomitant expression of HTR2C and its intragenic miRNAs is tempting to investigate.

miRIAD helped us to identify two interesting gene loci involved in complex human diseases with this quick and unpretentious gene survey. We speculate that many other crucial host/miRNA regulatory mechanisms could be revealed by taking advantage of using miRIAD for initial and/or advanced exploration.

## Discussion and conclusion

As the number of newly discovered miRNAs is constantly increasing, our understanding of the importance and the frequency of intragenic miRNAs has also been expanding (5, 10, 13, 15). For example, the past miRBase release 11 (April 2008) had around 47% of intragenic miRNAs (3), and this proportion increased to 53% in the miRBase 19 (August 2012) and to 57% in the miRBase (20). miRIAD was created to help dealing with the challenges of unraveling the functional relationships between miRNAs and their host genes.

miRIAD data are organized in five layers of information. The first layer contains annotation for protein-coding and miRNA genes, including the official gene name, gene aliases and annotation. The second layer provides genomic information for host and miRNAs. The third layer contains gene expression for miRNAs and coding genes and expression correlation between intragenic miRNAs and their host genes. The fourth layer includes miRNA target prediction information (providing binding sites as well). The fifth layer contains additional information, which extends to protein–protein interaction data for host genes as well as interaction partners that are targeted by host’s intragenic miRNA. Additionally, a set of useful external links to other databases is given. All these information are organized in a streamlined graphical web tool and full integrated into a MySQL relational database. For users who want to manipulate miRIAD information in a local environment, we provide links to download raw data and python code. Specific information not found in those files can be obtained upon request. Therefore, miRIAD can be used to investigate miRNAs in a very integrative context, with special attention to functional features, such as protein– protein interaction, miRNAs targeting host mRNAs or their partners in a functional network. We believe that our web tool can be used as a starting point for developing and testing new hypotheses related to miRNA gene regulation, for one gene or for large-scale data. Importantly, scripts have been developed and pipelined to deal with forthcoming updates.

miRIAD improvements, updates and further development will be ongoing. For example, we envision including additional species and other useful data, such as expression from unhealthy samples. Information on the last and upcoming updates can be found on the miRIAD website.

In conclusion, miRIAD provides a systematic, integrative, user-friendly, and easy-to-use platform to investigate inter- and intragenic miRNAs, host genes and their relationships for five species, including human and mouse. Users can query for and clearly retrieve miRNA and host gene information. Therefore, we believe that miRIAD can substantially improve the way in which we investigate intragenic miRNA and host genes.

## Supplementary Data

Supplementary data are available at *Database* Online.

Supplementary Data

## References

[1] Krol J. Loedige I. Filipowicz W. (2010) The widespread regulation of microRNA biogenesis, function and decay. Nat. Rev. Genet., 11, 597–610 20661255 10.1038/nrg2843

[2] Bartel D.P. (2009) MicroRNAs: target recognition and regulatory functions. Cell, 136, 215–233 19167326 10.1016/j.cell.2009.01.002 PMC3794896

[3] Kozomara A. Griffiths-Jones S. (2011) miRBase: integrating microRNA annotation and deep-sequencing data. Nucleic Acids Res., 39, D152–D157 21037258 10.1093/nar/gkq1027 PMC3013655

[4] Rodriguez A. Griffiths-Jones S. Ashurst J.L. (2004) Identification of mammalian microRNA host genes and transcription units. Genome Res., 14, 1902–1910 15364901 10.1101/gr.2722704 PMC524413

[5] Hinske L.C.G. Galante P.A.F. Kuo W.P. (2010) A potential role for intragenic miRNAs on their hosts' interactome. BMC Genomics, 11, 533 20920310 10.1186/1471-2164-11-533 PMC3091682

[6] Hinske L.C. Heyn J. Galante P.A.F. (2013) Setting up an intronic miRNA database. Methods Mol. Biol., 936, 69–76 23007499 10.1007/978-1-62703-083-0_5

[7] Baskerville S. Bartel D.P. (2005) Microarray profiling of microRNAs reveals frequent coexpression with neighboring miRNAs and host genes. RNA, 11, 241–247 15701730 10.1261/rna.7240905 PMC1370713

[8] Monteys A.M. Spengler R.M. Wan J. (2010) Structure and activity of putative intronic miRNA promoters. RNA, 16, 495–505 20075166 10.1261/rna.1731910 PMC2822915

[9] Yan L. Hao H. Elton T.S. (2011) Intronic microRNA suppresses endothelial nitric oxide synthase expression and endothelial cell proliferation via inhibition of STAT3 signaling. Mol. Cell. Biochem., 357, 9–19 21611796 10.1007/s11010-011-0870-x

[10] Kinoshita M. Ono K. Horie T. (2010) Regulation of adipocyte differentiation by activation of serotonin (5-HT) receptors 5-HT2AR and 5-HT2CR and involvement of microRNA-448-mediated repression of KLF5. Mol. Endocrinol., 24, 1978–1987 20719859 10.1210/me.2010-0054 PMC5417392

[11] Cho S. Jang I. Jun Y. (2013) MiRGator v3.0: a microRNA portal for deep sequencing, expression profiling and mRNA targeting. Nucleic Acids Res., 41, D252–D257 23193297 10.1093/nar/gks1168 PMC3531224

[12] Meyer L.R. Zweig A.S. Hinrichs A.S. (2013) The UCSC Genome Browser database: extensions and updates 2013. Nucleic Acids Res., 41, D64–D69 23155063 10.1093/nar/gks1048 PMC3531082

[13] Godnic I. Zorc M. Jevsinek Skok D. (2013) Genome-wide and species-wide in silico screening for intragenic MicroRNAs in human, mouse and chicken. PLoS One, 8, e65165 23762306 10.1371/journal.pone.0065165 PMC3675212

[14] Maselli V. Di Bernardo D. Banfi S. (2008) CoGemiR: a comparative genomics microRNA database. BMC Genomics, 9, 457 18837977 10.1186/1471-2164-9-457 PMC2567348

[15] He C. Li Z. Chen P. (2012) Young intragenic miRNAs are less coexpressed with host genes than old ones: implications of miRNA-host gene coevolution. Nucleic Acids Res., 40, 4002–4012 22238379 10.1093/nar/gkr1312 PMC3351155

[16] Brawand D. Soumillon M. Necsulea A. (2011) The evolution of gene expression levels in mammalian organs. Nature, 478, 343–348 22012392 10.1038/nature10532

[17] Meunier J. Lemoine F. Soumillon M. (2013) Birth and expression evolution of mammalian microRNA genes. Genome Res., 23, 34–45 23034410 10.1101/gr.140269.112 PMC3530682

[18] Kim D. Pertea G. Trapnell C. (2013) TopHat2: accurate alignment of transcriptomes in the presence of insertions, deletions and gene fusions. Genome Biol., 14, R36 23618408 10.1186/gb-2013-14-4-r36 PMC4053844

[19] Trapnell C. Salzberg S.L. (2009) How to map billions of short reads onto genomes. Nat. Biotechnol., 27, 455–457 19430453 10.1038/nbt0509-455 PMC2836519

[20] Trapnell C. Williams B.A. Pertea G. (2010) Transcript assembly and quantification by RNA-Seq reveals unannotated transcripts and isoform switching during cell differentiation. Nat. Biotechnol., 28, 511–515 20436464 10.1038/nbt.1621 PMC3146043

[21] Marco A. Griffiths-Jones S. (2012) Detection of microRNAs in color space. Bioinformatics, 28, 318–323 22171334 10.1093/bioinformatics/btr686 PMC3268249

[22] Robinson M.D. McCarthy D.J. Smyth G.K. (2010) edgeR: a Bioconductor package for differential expression analysis of digital gene expression data. Bioinformatics, 26, 139–140 19910308 10.1093/bioinformatics/btp616 PMC2796818

[23] Dill H. Linder B. Fehr A. (2012) Intronic miR-26b controls neuronal differentiation by repressing its host transcript, ctdsp2. Genes Dev., 26, 25–30 22215807 10.1101/gad.177774.111 PMC3258962

[24] Liu M. Roth A. Yu M. (2013) The IGF2 intronic miR-483 selectively enhances transcription from IGF2 fetal promoters and enhances tumorigenesis. Genes Dev., 27, 2543–2548 24298054 10.1101/gad.224170.113 PMC3861668

[25] Zhu Y. Lu Y. Zhang Q. (2012) MicroRNA-26a/b and their host genes cooperate to inhibit the G1/S transition by activating the pRb protein. Nucleic Acids Res., 40, 4615–4625 22210897 10.1093/nar/gkr1278 PMC3378857

[26] Radfar M.H. Wong W. Morris Q. (2011) Computational prediction of intronic microRNA targets using host gene expression reveals novel regulatory mechanisms. PLoS One, 6, e19312 21694770 10.1371/journal.pone.0019312 PMC3111417

[27] Pollak M. (2008) Insulin and insulin-like growth factor signalling in neoplasia. Nat. Rev. Cancer, 8, 915–928 19029956 10.1038/nrc2536

[28] da Cunha J.P.C. Galante P.A.F. de Souza J.E. (2009) Bioinformatics construction of the human cell surfaceome. Proc. Natl Acad. Sci. USA, 106, 16752–16757 19805368 10.1073/pnas.0907939106 PMC2757864

[29] de Souza J.E.S. Galante P.A.F. de Almeida R.V.B. (2012) SurfaceomeDB: a cancer-orientated database for genes encoding cell surface proteins. Cancer Immun., 12, 15 23390370 PMC3554024

[30] Gorvin C.M. Wilmer M.J. Piret S.E. (2013) Receptor-mediated endocytosis and endosomal acidification is impaired in proximal tubule epithelial cells of Dent disease patients. Proc. Natl Acad. Sci. USA, 110, 7014–7019 23572577 10.1073/pnas.1302063110 PMC3637698

[31] Mickey B.J. Sanford B.J. Love T.M. (2012) Striatal dopamine release and genetic variation of the serotonin 2C receptor in humans. J. Neurosci., 32, 9344–9350 22764241 10.1523/JNEUROSCI.1260-12.2012 PMC3431013

