# Supplementary material for: miRIAD—integrating microRNA inter- and intragenic data
Source: Database (Oxford). 2014 Oct 4;2014:bau099. doi: 10.1093/database/bau099 (PMC4186326; doi:10.1093/database/bau099)
Supplement: Supplementary Data [file supp_2014_bau099_index.html]

Supplementary Data 

# miRIAD—integrating **mi**cro**R**NA **i**nter- **a**nd intragenic **d**ata

## Supplementary Data

files

**Files in this Data Supplement:**

- Supplementary Data - zip file
